# Disseminated Infection With *Mycobacterium genavense* in the Setting of HIV Infection Misdiagnosed as Sarcoidosis

**DOI:** 10.1155/crdi/2249985

**Published:** 2025-08-24

**Authors:** Alireza Eskandari, Mitra Rezaei, Minoosh Shabani, Mihan Pourabdoullah, Afshin Moniri, Majid Marjani

**Affiliations:** ^1^Genomic Research Center, Shahid Beheshti University of Medical Sciences, Tehran, Iran; ^2^Clinical Tuberculosis and Epidemiology Research Center, National Research Institute of Tuberculosis and Lung Diseases (NRITLD), Shahid Beheshti University of Medical Sciences, Tehran, Iran; ^3^Department of Infectious Diseases and Tropical Medicine, Loghman Hakim Hospital, Shahid Beheshti University of Medical Sciences, Tehran, Iran; ^4^Infectious Diseases and Tropical Medicine Research Center, Shahid Beheshti University of Medical Sciences, Tehran, Iran; ^5^Chronic Respiratory Diseases Research Center, National Research Institute of Tuberculosis and Lung Diseases (NRITLD), Shahid Beheshti University of Medical Sciences, Tehran, Iran

**Keywords:** HIV, immunocompromised, *Mycobacterium genavense*, sarcoidosis

## Abstract

*Mycobacterium genavense* was first identified in a patient with HIV. Here, we describe a 40-year-old man with prolonged fever and mediastinal and abdominal lymphadenopathy, who was initially misdiagnosed with sarcoidosis. A molecular study was conducted after mycobacterium was isolated from a lymph node biopsy, leading to the identification of *M. genavense*. The identification of this microbe, along with recurrent oral candidiasis and varicella skin lesions, raised suspicion of an immunodeficiency disorder, which ultimately resulted in an HIV diagnosis. Concurrently, the patient experienced polyradiculopathy caused by cytomegalovirus. This case highlights that after identifying a granuloma in tissue, a comprehensive investigation to exclude infectious causes using microbiological and molecular methods is crucial.

## 1. Introduction

*Mycobacterium genavense* is a nontuberculous mycobacterium (NTM) which has been discovered in a human immunodeficiency virus (HIV)–infected individual for the first time in 1992 [1, 2]. Most infected cases are patients infected with HIV whose CD_4_ lymphocyte counts are less than 50 cells/mm^3^, and about 3.9%–12.8% of NTM infections among people living with HIV are attributed to these bacteria [3, 4]. In addition to HIV cases, other immunocompromised patients, including those who have lymphoproliferative malignancies or have undergone allogeneic stem cell transplantation and those who receive immunosuppressive drugs, are at greater risk for developing progressive infection with *M. genavense*. Nonetheless, immunocompetent individuals also could have these bacteria colonized in their gastrointestinal (GI) tract, and the acid-fast bacilli (AFB) could be detectable in even large amounts in feces despite no obvious signs and symptoms [2, 5]. Although *M. genavense* could be detectable in respiratory secretions, no person-to-person transmission has been reported yet. It has also been isolated from dogs, cats, monkeys, rabbits, and some kinds of birds [5]. Clinical presentations of affected patients are nonspecific and similar to those of *Mycobacterium avium complex*–infected individuals and range from lymphadenopathy, GI tract involvement, hepatosplenic disease, and bone marrow infiltration to disseminated forms [6, 7]. To proliferate this bacterium in culture samples, special preparations in solid media, including pH regulation, preserving temperature between 37°C and 45°C, and mycobactin J addition with a 3- to 12-week incubation period, should be considered. Hence, considering the slow growth pattern in the common mycobacterial culture media, diagnosis is usually difficult and needs high clinical suspicion and the recruitment of accurate molecular assays [8].

In this case report, we present a 40-year-old man who was admitted with severe abdominal pain, significant weight loss, and mediastinal and abdominal adenopathy; finally, disseminated *Mycobacterium genavense* was identified in the context of HIV infection.

## 2. Case Presentation

**Figure 1:**
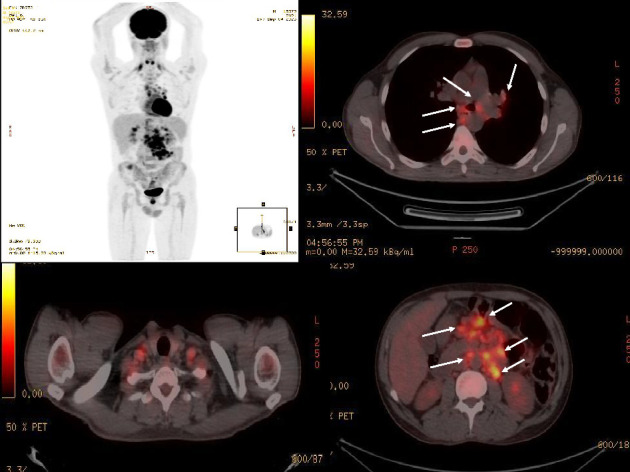
Lung, abdomen, and pelvis positron emission tomography (PET) scan; hypermetabolic mediastinal, left hilar, right retrocrural, and abdominal adenopathies are noted (arrows).

**Figure 2:**
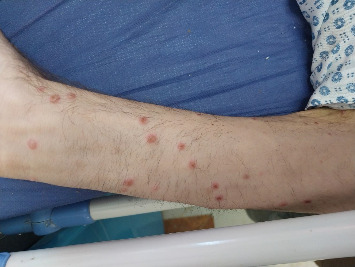
Disseminated cutaneous lesions, predominantly in the form of vesicles.

**Figure 3:**
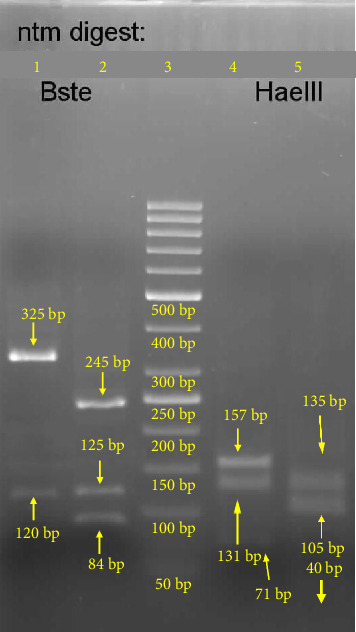
The restriction fragment length polymorphism pattern of the hsp65 amplicon from *Mycobacterium genavense*. Line 1: patient sample, fragment digested by BsteII. Line 2: positive control (*M. tuberculosis*) BsteII. Line 3: ladder. Line 4: positive control HaeIII. Line 5: patient sample, fragment digested by HaeIII.

**Figure 4:**
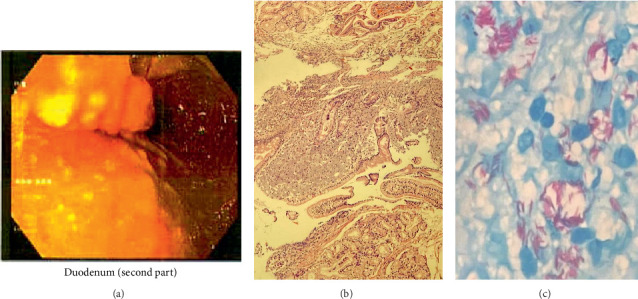
Upper gastrointestinal endoscopy and biopsy. (a) Multiple nodularities are seen in the second part of the duodenum. (b) Duodenal mucosa with subepithelial aggregates of histiocytes. (c) Countless acid-fast bacilli are highlighted by Ziehl–Neelsen stain.

A 40-year-old man was referred to our hospital due to recurrent fever, anorexia, severe abdominal pain, weakness of the lower limbs, significant weight loss (about 25 kg) over seven months, and positive mycobacterial culture related to transbronchial node aspiration (TBNA). The patient's symptoms started at first with oral lesions about 7 months ago, which were responsive to nystatin oral drops despite their recurrent pattern. Disseminated skin lesions with a predominantly bullous pattern and spontaneous but recurrent healing, anorexia, and intermittent fever also existed during this period. The weakness of the lower limbs had a progressive pattern in such a way that, despite being able to do his work by himself at first, the lower limb forces diminished to such an extent that walking was only possible with a cane. Generalized abdominal pain was initiated 1 month ago and has amplified during the last 7 days. He was married, a motor courier, a nonsmoker with no addiction, and with no remarkable past medical history. Before his admission to our hospital, he had several admissions in different centers with the impression of a fever of unknown origin (FUO). No obvious beneficial outcome was achieved during that period. Except for elevated levels of ESR (76 mm/h) and beta-2-microglobulin (3.8 mg/L) and positive rheumatoid factor, other hematologic investigations, including complete blood count and vasculitis blood markers, were within normal ranges. HIV serology was nonreactive by enzyme-linked immunosorbent Assay (ELISA) and equivocal by electrochemiluminescence (ECL) immunoassay, but no further investigation was performed. A serology test for *Brucella* was also negative. Abdominal sonography showed no pathognomonic findings, and echocardiography was normal. Lung compound topography showed a normal lung parenchymal pattern with mediastinal and bilateral hilar adenopathies. Punch biopsy of skin lesions was suggestive of hypersensitivity reactions. A positron emission tomography (PET) scan showed disseminated hypermetabolic mediastinal and abdominal adenopathies (Figure 1). During the past 3 months, and based on a left para-aortic lymph node core needle biopsy which exhibited granulomatous inflammation, he has undergone corticosteroid therapy with prednisolone (5 mg/day), concerning a possible diagnosis of sarcoidosis without any improvement. So, fiber optic bronchoscopy, bronchoalveolar lavage (BAL), and TBNA were performed. Microscopic investigation of BAL showed no evidence of either bacterial infection, but neutrophil-rich inflammatory cell infiltrations with AFB were seen in TBNA. Also, a re-examination of the core needle biopsy showed the AFB in tissue. Finally, he was referred to our hospital after mycobacterium grew in the Lowenstein medium from the fresh sample of TBNA after 19 days of incubation, and with the primary impression of tuberculosis (TB). No mycobacteria were isolated from the BAL sample after 70 days of incubation. In our center, his vital signs were within normal ranges. In general appearance, the patient was cachectic. On physical examination, multiple white oral patches were noted. The abdomen was soft with no tenderness, guarding, or palpable organomegaly. Lower limb forces had been reduced, and the skin appearance was significant for disseminated vesicular lesions (Figure 2). Hematologic investigations showed higher elevation of ESR (104 mm/h), and anemia (Hgb: 8 g/dl). Para-aortic lymph node core needle biopsy block was brought to our center for re-examination, and multiplex polymerase chain reaction (PCR) typing (PCR-RFLP targeting the hsp65 gene) was performed by the molecular pathology section. The result was consistent with *M. genavense* (Figure 3), and treatment with rifampin (450 mg/d), ethambutol (800 mg/d), azithromycin (250 mg/d), and amikacin (500 mg/d) was initiated. Serum immunoglobulin levels were normal. Dihydrorhodamine (DHR) flow cytometry test ruled out chronic granulomatous disease (CGD). Flow cytometry was done and showed very low serum CD_4_ lymphocyte counts (CD_4_ < 5 cells/mm^3^) and also decreased the number of CD_19_ (8 cells/mm^3^) and CD_20_ (7 cells/mm^3^) lymphocytes, so an HIV Ag/Ab combination immunoassay (Abbott ARCHITECT HIV Ag/Ab Combo) was performed. HIV antibody was detected, and serum HIV viral load was 48,720 IU/mm^3^. PCR test from skin lesions was positive for the varicella-zoster virus (VZV) (targeting the glycoprotein E gene of the virus), so acyclovir was initiated. Electromyography and nerve conduction velocity (EMG-NCV) were performed, which indicated distal symmetrical axonal sensorimotor polyneuropathy. A lumbar puncture was performed. Cerebrospinal fluid (CSF) and simultaneous serum sugar levels were 40 and 87 mg/dL, respectively, with no pleocytosis. CSF adenosine deaminase was 31 U/L, but mycobacterium DNA was not detected. Nonetheless, due to high levels of cytomegalovirus (CMV) viral load in CSF (4700 IU/mL), accompanied by lower limb muscle weakness and with the diagnosis of CMV radiculopathy, acyclovir was changed to ganciclovir. Due to severe abdominal pain, an upper GI endoscopy was performed, which showed multiple nodules at the second portion of the duodenum. The presence of granuloma formation and AFB in pathologic investigation (Figure 4) and the detection of *M. genavense* in molecular assays made the diagnosis of GI mycobacterial infection certain. During the hospitalization period and in addition to the antibacterial regime for *M. genavense*, the patient underwent antiretroviral therapy (ART) with dolutegravir (50 mg twice a day), emtricitabine/tenofovir disoproxil fumarate (200 mg/300 mg daily), and ganciclovir, and also antifungal treatment with fluconazole (400 mg/day). After about 60 days of treatment, the general condition of the patient improved, and after replacing ganciclovir with valganciclovir, the patient was discharged with the above-mentioned treatment except for amikacin. After receiving 12 months of multidrug therapy with antimycobacterial and antiviral agents, HIV and CMV serum viral loads were undetectable, and general condition significantly improved. He had gained weight and no longer needed a cane to walk. Furthermore, improvement of GI symptoms was promising, and follow-up upper GI endoscopy demonstrated a relatively healing pattern. However, despite a gradual augmentation in serum CD_4_ cell absolute counts, it generally remained low so that the highest level did not exceed 50 cells/mm^3^. We plan to continue antimycobacterial therapy and valganciclovir until reaching the CD_4_ cell counts of at least 100 and they are stabilized for at least 3 months.

## 3. Discussion

As an opportunistic nontuberculous mycobacterium, *M. genavense* predominantly involves the GI tract [4]. In patients infected with HIV, a significant decrease in CD_4_ lymphocyte counts plays a critical role in disease occurrence. Routine mycobacterial cultures are usually negative for these bacteria. In case of AFB detection on smear or histopathological finding accompanied by negative PCR for *M. tuberculosis*, molecular diagnostic techniques, including 16s rRNA sequence analysis or gene sequencing for timely diagnosis, should be done [6]. To the best of our knowledge, this is the fourth case of *M. genavense* reported in Iran. Just like the previous cases, this patient also presented with GI and constitutional symptoms. One of the reported cases also had central nervous system involvement, and further investigations revealed that all of them had an immunocompromised state caused by HIV infection [9, 10].

In this patient, initially due to suspicion of sarcoidosis, prednisolone was initiated. This diagnosis was probably made due to mediastinal and bilateral hilar adenopathies and the appearance of granulomas in the lymph node biopsy. Sarcoidosis, as a systemic inflammatory disease, is usually diagnosed based on radiologic, clinical, and histopathologic findings, along with the exclusion of other causes, especially mycobacterial infections. Similar cases have been reported in the literature that was initially misdiagnosed as sarcoidosis [7, 11, 12]. This may be explained by the challenges in isolating *M. genavense*. This infection has also been reported in people who have had sarcoidosis for many years. It appears that immunosuppressive treatment has made them susceptible to this opportunistic infection. [13, 14]. In a systematic review of *M. genavense* infection in non-HIV immunocompromised hosts by Mahmood et al., published in 2017, 14% of them had sarcoidosis as an underlying condition [6]. On the other hand, the potential role of mycobacterial infection in the pathogenesis of sarcoidosis has been suggested by molecular studies [15]. This case was referred to our hospital as a referral TB center after the growth of mycobacteria in the fresh specimen of TBNA. Although TB is still the most common mycobacteria in our setting, we should do our best to identify the type of mycobacterium, because the treatment can be completely different. Molecular typing assay (PCR-RFLP targeting the hsp65 gene using primers Tb11: 5′-ACC AAC GAT GGT GTG TCC AT-3′ and Tb12: 5′-CTT GTC GAA CCG CAT ACC CT-3′ as described by others [16]) confirmed *M. genavense*. This method is fast, nonexpensive, well established, and widely used for accurate identification and differentiation of nontuberculous mycobacterium, including *M. genavense* [17]. Also, he had recurrent episodes of spontaneously healing skin lesions and oral thrush. These symptoms should have raised the suspicion of immunodeficiency much earlier, and a detailed investigation should have been done in this regard. We performed flow cytometry for B and T cell markers, including CD_3_, CD_4_, CD_8_, CD_19_, CD_20,_ and CD_16+56_, DHR test to rule out CGD, and measured the level of serum immunoglobulins. In addition, we considered HIV infection as a common cause of acquired immunodeficiency. The patient had previously been tested for HIV, and although the ECL HIV test had an equivocal result, unfortunately, no further investigation was performed. HIV PCR testing is essential when a patient's symptoms can be explained by HIV infection (especially in the presence of an unusual and opportunistic pathogen), but serology is negative or inconclusive.

The immunodeficient state of the patient in the context of neglected HIV infection justified the patient's signs and symptoms even more. A well-formed granuloma had been found in the abdominal lymph node, but a specimen taken about 3 months later by TBNA revealed that the lymph node contained numerous AFB without granuloma, probably due to worsening immunodeficiency.

*M. genavense* tends to colonize the GI tract. In a meta-analysis containing 223 patients, including both HIV- and non-HIV–infected patients, abdominal pain and hepatosplenomegaly were among the most common manifestations [18]. In our patient, due to severe abdominal pain and anorexia, upper GI endoscopy was performed, and the results were consistent with *M. genavense*.

Lungs are another place where *M. genavense* may involve. It is usually determined with cavitary lesions and reticulonodular patterns on imaging. Although isolated pulmonary infection could occur, nevertheless, and in concordance with what we described here, we expect disseminated disease in patients infected with HIV with severely immunocompromised states whose CD_4_ cell counts range between 10 and 83 cells/mm^3^ [19]. Despite mediastinal lymphadenopathy in our patient, there were no signs of lung parenchymal involvement.

Infection with *M. genavense* could present with seizure, gait abnormality, and signs and symptoms of meningitis due to invasion of the central nervous system, proliferating in CSF, and leading to cerebral mass. Nonetheless, only a few cases have been identified so far, which were almost all among HIV patients [10, 20, 21]. Neurological examinations of our patient, except for progressive lower limb paresis, were unremarkable. CSF analysis showed no pleocytosis, and no mycobacterium DNA was identified in CSF. However, CMV was detected, which justified the neurological symptoms.

Determining which drug combination could be more effective in treating *M. genavense,* due to the challenging antibacterial susceptibility testing and lack of clinical trials, is difficult [6]. However, it has been demonstrated that macrolide-containing regimes lead to better outcomes [18]. Hence, it has been recommended that multidrug therapy (minimally three drugs) with macrolide, ethambutol, and rifampin as first line, and amikacin, levofloxacin, or clofazimine as alternatives for at least 12–18 months (12 months after culture conversion) could be an effective strategy [22].

Due to the underlying immunodeficiency state of these patients, the prognosis is relatively poor [8]. Hence, an increase in CD_4_ cell count and subsequent immune system improvement could lead to a better prognosis. Fortunately, the 1-year follow-up of our patient showed relatively promising results in terms of general condition improvement. It has been shown in a cohort study performed on HIV and *M. genavense* coinfected cases that the mean survival rate in patients under ART was 70.4 months, which was significantly higher compared to patients who did not receive ART [23].

## 4. Conclusion

This case reminds us that after finding a granuloma in the tissue, all necessary steps should be taken to rule out infectious causes, such as specific stains, cultures, and molecular diagnostic studies. Also, if a mycobacterium is isolated, an attempt should be made to determine its type. *M. genavense* is a rare infection whose discovery should be a stimulus for examining the immune system. Patients infected with HIV are at great risk for developing opportunistic pathogens, including NTM, predominantly in the form of disseminated disease.

## Data Availability

The data that support the findings of this study are available on request from the corresponding author.
